# The phylogeographic structure of *Hydrilla verticillata* (Hydrocharitaceae) in China and its implications for the biogeographic history of this worldwide-distributed submerged macrophyte

**DOI:** 10.1186/s12862-015-0381-6

**Published:** 2015-05-24

**Authors:** Jinning Zhu, Dan Yu, Xinwei Xu

**Affiliations:** National Field Station of Freshwater Ecosystem of Liangzi Lake, College of Life Sciences, Wuhan University, Wuhan, PR China

**Keywords:** Biogeography, China, *Hydrilla*, Phylogeographic structure, Submerged macrophyte, *trn*L-F

## Abstract

**Background:**

Aquatic vascular plants are a distinctive group, differing from terrestrial plants in their growth forms and habitats. Among the various aquatic plant life forms, the evolutionary processes of freshwater submerged species are most likely distinct due to their exclusive occurrence in the discrete and patchy aquatic habitats. Using the chloroplast *trn*L-F region sequence data, we investigated the phylogeographic structure of a submerged macrophyte, *Hydrilla verticillata*, the single species in the genus *Hydrilla*, throughout China, in addition to combined sample data from other countries to reveal the colonisation and diversification processes of this species throughout the world.

**Results:**

We sequenced 681 individuals from 123 sampling locations throughout China and identified a significant phylogeographic structure (N_ST_ > G_ST_, *p* < 0.01), in which four distinct lineages occurred in different areas. A high level of genetic differentiation among populations (global F_ST_ = 0.820) was detected. The divergence of *Hydrilla* was estimated to have occurred in the late Miocene, and the diversification of various clades was dated to the Pleistocene epoch. Biogeographic analyses suggested an East Asian origin of *Hydrilla* and its subsequent dispersal throughout the world.

**Conclusions:**

The presence of all four clades in China indicates that China is most likely the centre of *Hydrilla* genetic diversity. The worldwide distribution of *Hydrilla* is due to recent vicariance and dispersal events that occurred in different clades during the Pleistocene. Our findings also provide useful information for the management of invasive *Hydrilla* in North America.

**Electronic supplementary material:**

The online version of this article (doi:10.1186/s12862-015-0381-6) contains supplementary material, which is available to authorized users.

## Background

Aquatic vascular plants are a distinctive group, differing from terrestrial plants in their growth forms and habitats. They have multiple evolutionary origins from terrestrial environments and show a complex evolutionary history [1,2]. Many genera and species of aquatic plants are distributed worldwide [3,4]. Recently, the historical biogeographic scenarios of some genera, including their areas of origin and dispersal routes, have been inferred in the context of phylogenetics based on molecular evidence (e.g., [5–9]). However, the biogeographic history of cosmopolitan species of aquatic plants is seldom studied and needs to be explored through phylogeographic studies based on a broader sampling scheme.

An exponential growth of plant phylogeographic studies has been observed in Europe and North America in the past two decades, and a similar trend has recently been found in China and adjacent regions [10,11]. Common genetic discontinuities, locations of refuges, and routes of colonisation have been revealed in some regions by comparing phylogeographic structures among species [11–19]. In these plant phylogeographic studies, the majority of surveys were conducted on tree species and terrestrial plants, whereas studies on aquatic plants have been relatively scarce [10]. These studies on aquatic plants focused primarily on two groups: seagrasses (e.g., [20–22]) and emergent macrophytes in freshwater environments (e.g., [23–27]). Few studies have focused on freshwater submerged species (but see [28,29]), whose evolutionary processes are most likely distinct from those of emergent species due to their occurrence in exclusively aquatic habitats [2,30], and seagrasses due to their lower population connectivity in discrete and patchy habitats [31]. Therefore, phylogeographic studies on freshwater submerged macrophytes will provide new insights to increase our understanding of plant evolution.

Here, we focus on the submerged plant genus *Hydrilla*, a monotypic genus of the family Hydrocharitaceae, which is distributed worldwide. The single *Hydrilla* species *H. verticillata* (L.f.) Royle (hydrilla) is found on all continents except Antarctica [32,33]. This species is native to Asia, but it is uncertain whether it is truly native to Europe, Australia and Africa. Hydrilla was first recorded in North America from 1960 and South America from 2005 [4,32,34]. Similar to most aquatic plants, hydrilla possesses a variety of reproductive strategies to ensure its growth and establishment, including reproduction through seeds, fragmentation, turions on leaf axils and tubers (subterranean turions) [35]. Hydrilla grows in various types of aquatic habitats (such as lakes, rivers and ponds) from tropical to temperate regions, and apparent morphological and karyological variations have been observed in different populations worldwide [32,36–40]. Monoecious and dioecious strains and diploid, triploid and tetraploid plants have also been reported in hydrilla [32,37]. Furthermore, high levels of genetic differentiation have been revealed among worldwide samples of hydrilla based on isoenzyme patterns [37,38], random amplified polymorphic DNA (RAPD) profiling [41,42], and DNA sequences [40,43]. However, previous investigations did not include a sufficient number of samples to explore the evolutionary processes of this submerged species worldwide.

In this study, we first examined the phylogeographic structure of an extensive sample population of hydrilla from China using sequences of the chloroplast *trn*L-F region. We then inferred the biogeographic history of hydrilla by combining the *trn*L-F sequences from previous studies conducted worldwide. Our objectives were (1) to examine the genealogical patterns of hydrilla in China, and (2) to infer the original area and dispersal route of hydrilla. This study will provide a good example for us to understand the evolutionary processes that occur in submerged macrophytes.

## Results

### Genetic variation and phylogeographic structure

A total of 681 sequences were obtained, with lengths ranging from 1,066 to 1,105 bp. The length of the aligned sequences was 1,130 bp, and 32 polymorphic sites were observed, including 18 indels and 14 base substitutions. The sequences were collapsed into 9 haplotypes (A1, A2, B1–B4, C1, C2, and D1). The two most common haplotypes were C1 (occurring 359 times; 52.7 %) and B1 (occurring 227 times; 33.3 %), which were present in 78 populations and 49 populations, respectively. Haplotypes D1, B4, A1, and B3 were detected in 11, 6, 5 and 2 populations, respectively. The remaining three haplotypes, A2, B2, and C2, were each present in a single population. Of the 123 populations we surveyed, 93 populations were monomorphic and consisted of a single haplotype. In the nine groups we defined based on river basins, the highest diversity was present in the Yangtze River Basin (Hd = 0.625, Pi = 0.0038) and the river basins in Southeast China (Hd = 0.730, Pi = 0.0037) (Additional file 1). However, no polymorphism was detected in the three river basins located in Northeast China and North China. The Hd and Pi values for all of the populations surveyed were 0.608 and 0.0038, respectively (Additional file 1).

**Fig. 1:**
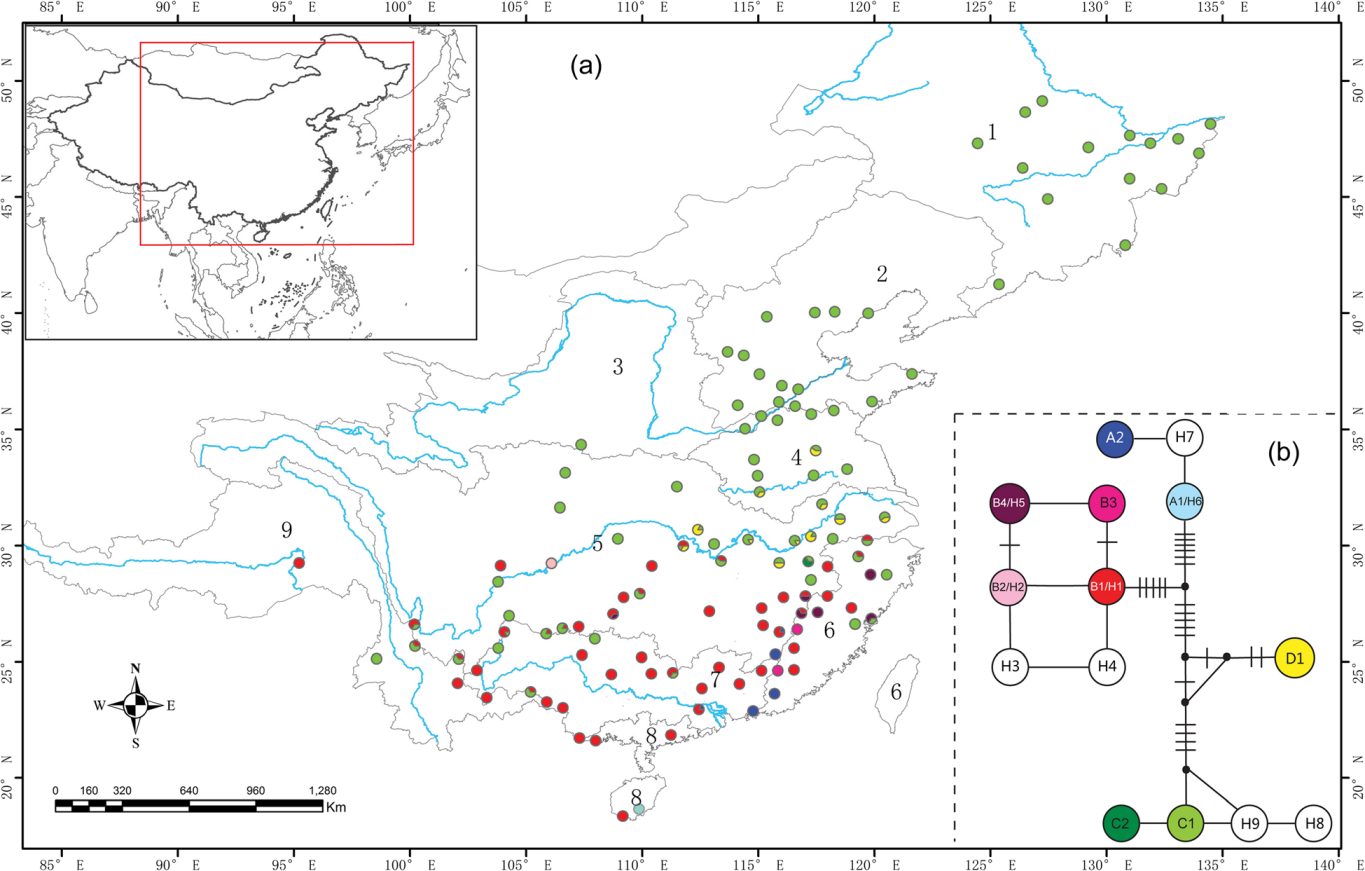
Sampling sites of *Hydrilla* populations and geographical distribution of cpDNA haplotypes. **a**) Sampling sites of 123 *Hydrilla* populations and geographical distribution of nine haplotypes of *trn*L-F sequences in China. The numbers 1-9 indicate nine main river basins: the Amur-Heilong River Basin (RB1), the Liao River and Hai River Basin (RB2), the Yellow River Basin (RB3), the Huai River Basin (RB4), the Yangtze River Basin (RB5), river basins in Southeast China (RB6), the Pearl River Basin (RB7), river basins in South China (RB8), and river basins in Southwest China (RB9). **b**) Haplotype network of the chloroplast *trn*L-F region. The haplotypes without colour are not found in China. Black dots and short lines represent inferred interior nodes that were absent in the samples

A haplotype network was constructed with four distinct groups based on all haplotypes from worldwide samples (see below for the definition of haplotypes) (Fig. 1b). The first group included four haplotypes (C1, C2, H8 and H9) and two Chinese haplotypes (C1 and C2) involved all of the basins except for RB8 in South China, whereas the frequency of occurrence was low in the three basins (RB6, RB7, and RB9) located in the southern part of China. The second group only included one haplotype, D1, and was detected in two basins (RB4 and RB5). In RB5, the second group was restricted to the middle and lower reaches of the Yangtze River. The third group consisted of six haplotypes (B1–B4, H3 and H4), of which four Chinese haplotypes (B1–B4) occurred mostly south of the Yangtze River in five basins (RB5–RB9). The fourth group comprised haplotypes A1, A2 and H7, of which two Chinese haplotypes (A1 and A2)were only found in six populations located in south and southeast China in four basins (RB5–RB8) (Fig. 1a). A permutation test showed that N_ST_ (0.842) was significantly greater than G_ST_ (0.799, P < 0.01), indicating that closely related haplotypes tended to occur in the same area. An AMOVA revealed that 17.97 % of the total variation occurred within populations, and 82.03 % occurred between populations. The global F_ST_ value (0.820) indicated a significant genetic structure among the 123 hydrilla populations. When we grouped the populations into basins, an AMOVA showed that large amounts of variation occurred both among the basins (40.09 %) and among the populations within basins (43.14 %) and that 16.77 % of the variation occurred within populations. The mismatch distribution of the overall populations was multimodal (not shown), and a sudden expansion model for hydrilla was therefore rejected.

### Phylogenetic relationships

**Fig. 2:**
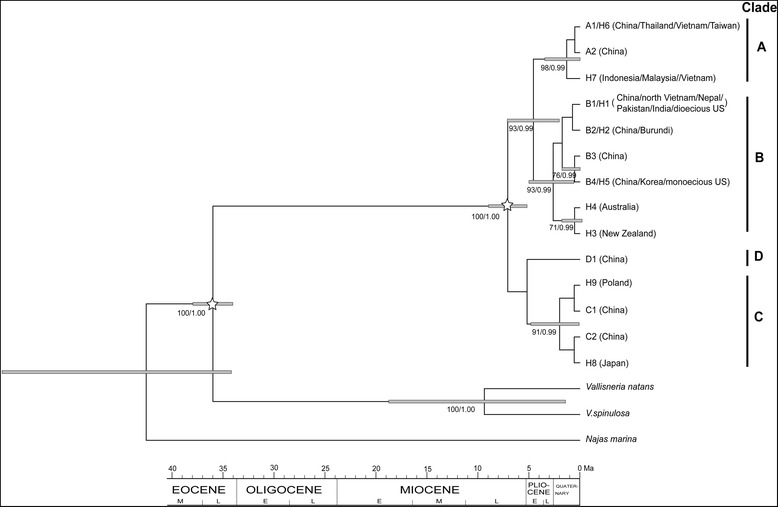
Chronogram of *Hydrilla* inferred from *trn*L-F sequences using BEAST. Two clade constraints are indicated with blank asterisks. Grey boxes indicate the 95 % highest posterior density intervals. The maximum likelihood bootstrap values of 70 and above (left) and the posterior probabilities of 0.95 and above (right) are shown at the nodes

The accessions collected from worldwide by Madeira *et al*. [43] were collapsed into 9 haplotypes: H1 (including samples from China, north Vietnam, Nepal, Pakistan, and India and dioecious US), H2 (Burundi), H3 (New Zealand), H4 (Australia), H5 (Korea and monoecious US), H6 (Thailand, Vietnam, and Taiwan), H7 (Indonesia, Malaysia, and Vietnam), H8 (Japan), and H9 (Poland). Four of the haplotypes were the same as haplotypes identified in our samples: H1 = B1, H2 = B2, H5 = B4, and H6 = A1. Thus, a total of 14 haplotypes were obtained and employed for phylogenetic reconstruction using three outgroups. ML analysis and Bayesian inference produced a similar topology (Fig. 2). The monophyly of hydrilla was strongly supported by both analyses (bootstrap support (BS) = 100 %, posterior probability (PP) = 1.00). Four distinct clades with robust support were revealed among the 14 haplotypes of hydrilla, consistent with four groups of the haplotype network. Haplotypes of the A lineage (A1/H6, A2, and H7) were located in south and Southeast China and Southeast Asia. B lineage haplotypes (B1/H1, B2/H2, B3, B4/H5, H3, and H4) were present in Korea, south of the Yangtze River in China, South Asia, Burundi, Australia and North America. The A and B lineages grouped into a clade with high support values (BS = 88 %, PP = 1.00). C lineage haplotypes (C1, C2, H8 and H9) were found in most areas of China, Japan and Poland. The D lineage haplotype (D1) was present only in China, showing a narrow range (Figs. 1a and 2).

### Divergence time estimates

**Fig. 3:**
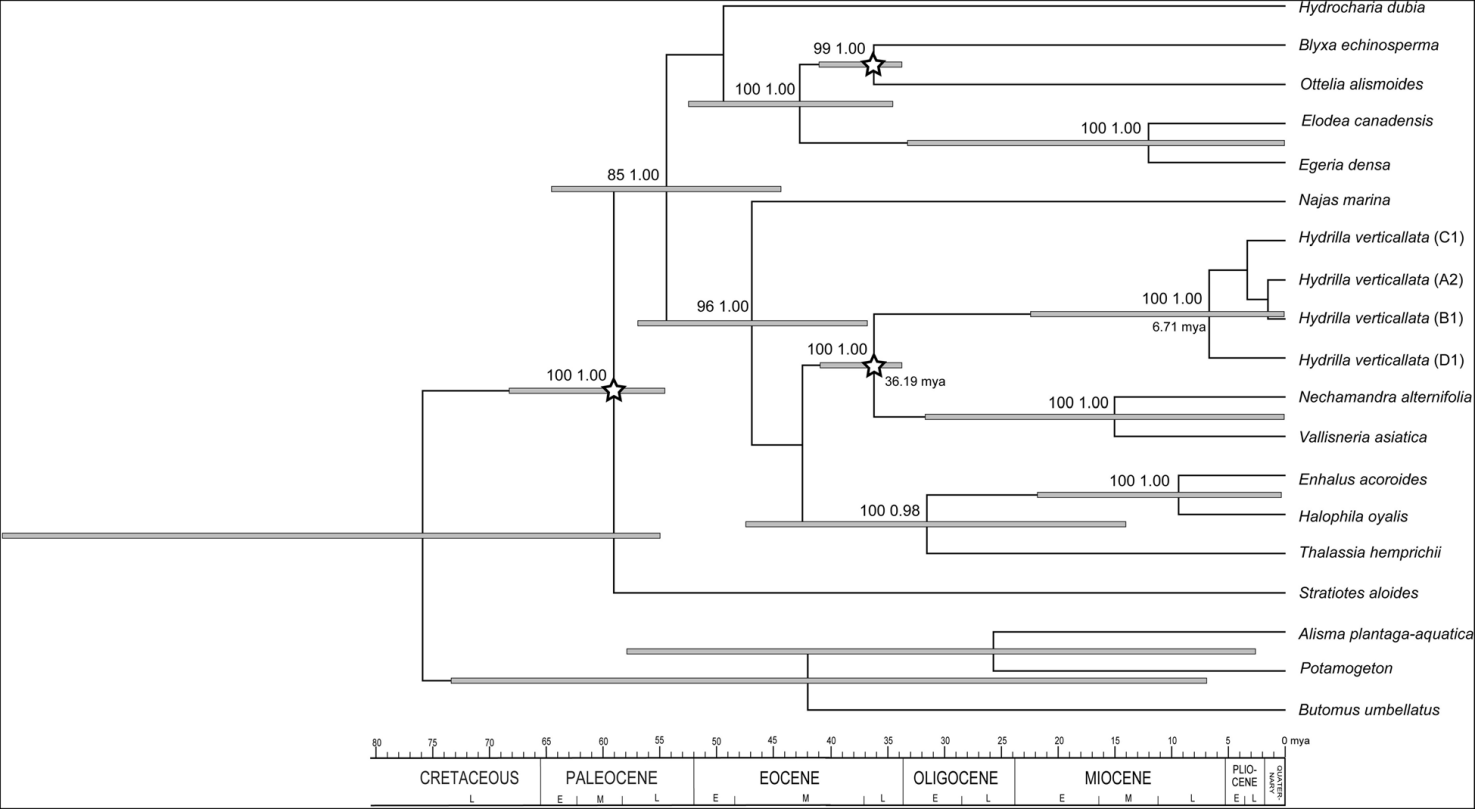
Chronogram of Hydrocharitaceae inferred from combined 18S + *rbc*L + *mat*K + *trn*K 5’ intron + *rpo*B + *rpo*C1 + *cob* + *atp*1 sequence data using BEAST. Three calibration points are indicated with blank asterisks. Grey boxes indicate 95 % highest posterior density intervals. The maximum likelihood bootstrap values of 70 and above (left) and the posterior probabilities of 0.95 and above (right) are shown at the nodes

The stem and crown ages of hydrilla were estimated to be 36.19 Ma (95 % HPD: 33.74–40.93 Ma) and 6.71 Ma (0.12–22.42 Ma), respectively, based on the combined data (Fig. 3). Due to no bootstrap support for the internal nodes of hydrilla in the multigene tree, which was caused by low polymorphism (Additional file 2), the *trn*L-F sequences were used to estimate interior divergence times. The crown age of clade A + B was estimated to be 4.57 Ma (95 % HPD = 2.06–7.15 Ma) based on the *trn*L-F sequence data (Fig. 2). The crown node ages of clades A, B and C were dated to 1.31 Ma (95 % HPD: 0.01–3.45 Ma), 2.63 Ma (0.59–4.99 Ma) and 2.00 Ma (0.09–4.79 Ma), respectively (Fig. 2).

### Historical biogeography inference

**Fig. 4:**
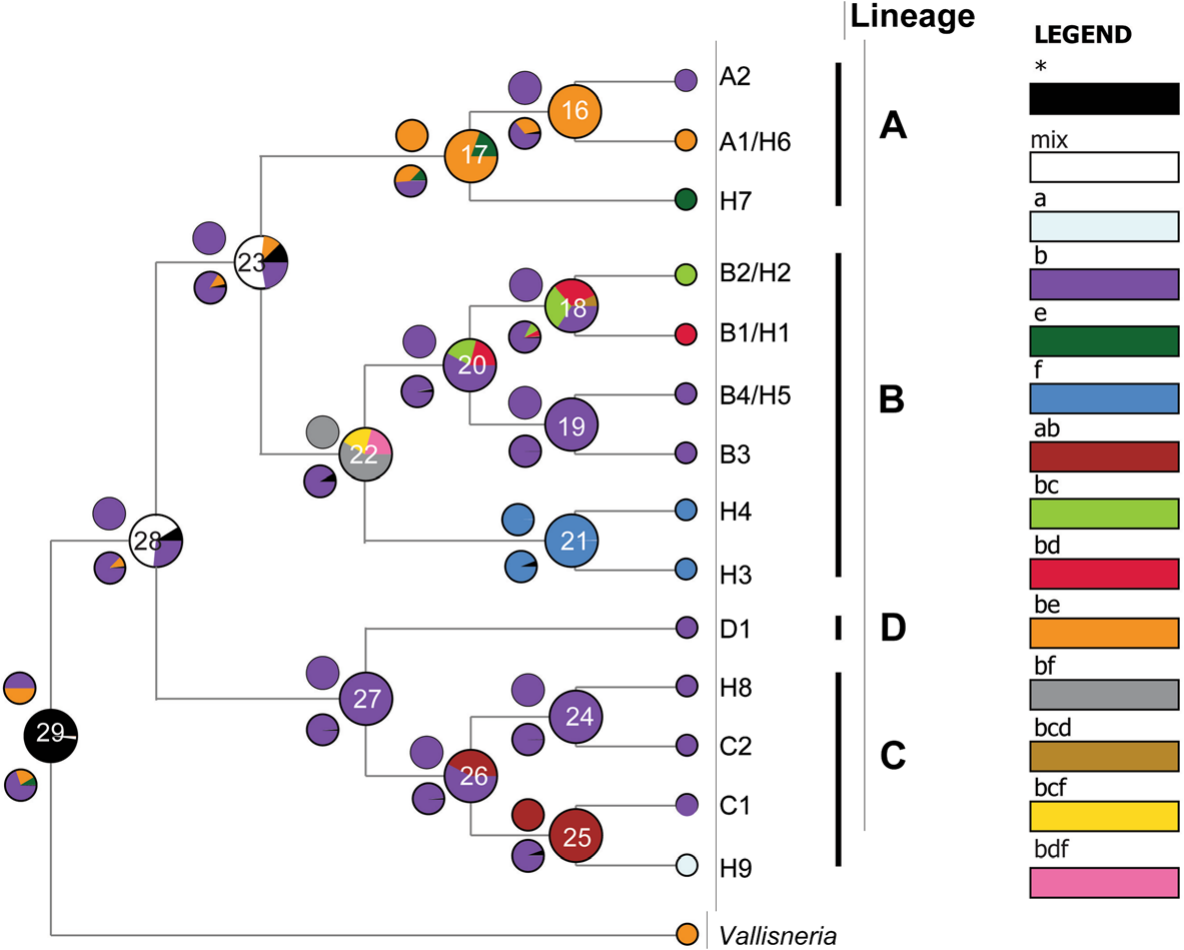
Reconstruction of the ancestral area of *Hydrilla*. The pie charts at each node were obtained using DEC analysis, and the smaller pie charts above and below each node were obtained through S-DIVA and BBM analysis, respectively. The colours correspond to possible ancestral areas; black with an asterisk represents other ancestral ranges; and white with “mix” indicates too many possible ancestral areas to determine. Lowercase letters represent different regions: **a**) Europe (Poland); **b**) East Asia (China/Korea/Japan); **c**) Africa (Burundi); **d**) South Asia (India/Nepal); **e**) Southeast Asia (Vietnam/Thailand/Malaysia/Indonesia); **f**) Oceania (Australia/New Zealand)

The S-DIVA, BBM and DEC analyses conducted with different numbers of maximum area settings obtained similar biogeographic inferences, with the exception of several nodes, and only the results for the 4 maximum areas are presented. The S-DIVA, BBM and DEC analyses all suggested that the ancestral area of hydrilla at node 28 was East Asia with the highest likelihood. For the ancestral range of interior node 23 of clade A + B and node 26 of clade C, East Asia was supported by all three analyses with the highest likelihood. At node 17 of clade A and node 22 of clade B, East Asia was supported as the ancestral range by BBM, whereas East Asia plus Southeast Asia and East Asia plus Oceania were inferred by S-DIVA and DEC, respectively (Fig. 4). Dispersal and vicariant events were also revealed. All the three analyses postulated vicariant events at nodes 17, 22 and 25. The dispersal events were detected at nodes 16, 18, 23, 26 in S-DIVA analysis, at nodes 16, 17, 22, 25 in BBM analysis and at nodes 16, 17, 18, 23, 26 in DEC analysis.

## Discussion

### Genetic variation in hydrilla

At the population level, more than three-fourths of the populations are composed of only a single haplotype. Although the percentage may be overestimated due to limited samples of each population and a single cpDNA fragment used, lack of intra-population variation seems frequent. This is most likely attributed to the strong ability of hydrilla to reproduce asexually. Hydrilla populations can expand rapidly via various vegetative propagules, including plant fragmentation, turions and tubers [44–46]. As vegetative reproduction is common in aquatic plants, modest variations within populations have also been observed in other species, e.g., *Ranunculus bungei* [29], *Podostemum ceratophyllum* [47], and *Hippuris vulgaris* [48]. Moreover, certain studies based on nuclear markers suggested that founder effects played an important role in the establishment of populations in aquatic plants [26,49,50]. To explore the role of founder effects in shaping population structure of hydrilla, further studies using nuclear markers are needed.

This study suggests that China is most likely the central area of genetic diversity for hydrilla. Both Madeira *et al*. [43] and Benoit [40] identified three clades from worldwide samples of hydrilla based on chloroplast *trn*L-F sequences, corresponding closely to those identified by Madeira *et al*. [41,42] using RAPD. As China is the geographic centre of the distribution range of hydrilla, the small number of accessions from China (less than 5 individuals) included in these studies is insufficient. Combined with the *trn*L-F sequences of samples obtained worldwide, our results revealed four clades in hydrilla, and China was the only area in which haplotypes from all four clades occurred (Fig. 2). Furthermore, a low level of genetic variation was observed in hydrilla samples from other areas, e.g., in Europe, nearly identical isoenzyme patterns were observed in plants from Ireland and Poland [51] and the same *trn*L-F sequences were present in plants from Ireland and Latvia [40]; in Africa, similar isoenzyme patterns or genetic types were revealed in plants from Uganda, Rwanda and Burundi [38,52]; in South Asia, samples from Nepal, Pakistan and India grouped into the same cluster according to random amplified polymorphic DNA (RAPD) analysis [41] and exhibited the same *trn*L-F sequences [43]; in Southeast Asia, individuals from Vietnam, Thailand, Malaysia and Indonesia grouped into the same cluster according to RAPD analysis [42] and included two clades of *trn*L-F sequences [43], Fig. 2; and in Australia, samples from five localities included two clades of *trn*L-F sequences [40]. Based on 109 samples from various areas in Asia and the Indo-Pacific region, the highest genetic diversity was found in China with microsatellite markers [52]. Therefore, the highest genetic diversity of hydrilla was most likely detected in populations from China.

### Phylogeographic structure of hydrilla

An important characteristic of hydrilla is the high value of global F_ST_ (0.820), indicating that most genetic variation is found among populations. The results of an AMOVA suggested that half of the detected genetic variations should be ascribed to genetic differentiation among the basins. This finding was supported by the significant phylogeographic structure revealed, in which the haplotypes of lineage C mostly occurred in the northern part of China, lineage B occurred in the southern part of China, lineage D haplotype was restricted to the Huai River and the middle and lower reaches of the Yangtze River, and lineage A was restricted to the southeast corner of China (Fig. 1). According to the distribution of haplotypes in basins, the isolation of individual basins appears to constitute a barrier to inter-basin gene flow. High genetic differentiation among basins was also reported in some species of aquatic plants, e.g., *Batrachium bungei* [49], *Podostemum irgangii* [53], and *Podostemum ceratophyllum* [47]. However, unlike these species, hydrilla can form tubers, which may survive for several days after removal from water [54] and remain viable even after ingestion and regurgitation by waterfowl [55]. It is possible that waterfowl migration could transport viable tubers across water basins. The high genetic differentiation of hydrilla populations observed among basins may be attributed to other factors associated with the process of colonisation. Two phylogeographic studies on aquatic plants with extensive sampling have been conducted in China (*Zizania latifolia* [26] and *Sagittaria trifolia* [25]). Similarly, no significant phylogeographic structure was revealed in these two species, and the highest diversity was reported from Northeast China, a finding that is different from hydrilla’s pattern. Because the two species are emergent and their evolutionary processes are likely to differ significantly from submerged groups [2], it is necessary to conduct comparative phylogeographic studies focusing on submerged macrophytes.

### Biogeographic history of hydrilla

The divergence time estimates for hydrilla showed a remarkably long branch between the stem node of this genus in the late Eocene and its crown node in the late Miocene (Figs. 2 and 3), suggesting long-term stasis or extinct lineages before diversification in hydrilla, similar to what has been found in several other genera of Hydrocharitaceae, such as *Najas*, *Ottelia*, and *Blyxa* [9]. The East Asian origin of hydrilla inferred through ancestral area reconstruction is supported by the fact that China is most likely the centre of genetic diversity for this genus. The first diversification of hydrilla into three lineages (clade A + B, clade C and clade D) was dated to the late Miocene (Fig. 3) and may have been triggered by the existence of warm-cool alternations and a cold, dry climate due to strengthened East Asian winter monsoons during the late Miocene and Pliocene [56–59]. Clade A + B dispersed from East Asia to other areas and then diverged into clades A and B during the early Pliocene (Fig. 2). The diversification events for clades A, B and C were all dated to the Pleistocene epoch, associated with Quaternary glacial/interglacial cycles. Due to the interior divergence times in hydrilla were estimated by the *trn*L-F sequences despite its high polymorphism (Additional file 2), their accuracy needed to be further tested by more sequence data.

The three analyses (S-DIVA, BBM and DEC) inferred vicariance at nodes 17, 22 and 25, of which node 17 is the crown node of clade A and 22 is the crown node of clade B with robust support (Fig. 2), indicating a vicariant event during the diversification in clade A and in clade B. The crown ages of both clades were dated to the Pleistocene epoch, in which cool glacial periods and warm interglacial periods were alternative. Climate change could be responsible for the vicariant events (e.g., [27]). For example, in clade A East Asian populations could have been isolated from Southeast Asian ones during more than one glacial period due to the emergence or disappearance of large land areas between these two areas caused by great sea level fluctuation [60]. The S-DIVA, BBM and DEC analyses inferred dispersal events at different nodes except node 16. Most dispersal events were relatively recent, suggesting that waterfowl dispersals are likely given transoceanic distribution. Waterfowl are considered the most significant dispersal agents for aquatic plants [61,62]. Although the ability of seeds to maintain viability after passing through the gut has been reported in some groups of submerged macrophytes, such as *Potamogeton* and *Najas* [63,64], whether hydrilla seeds can survive digestion by waterfowl has not been tested thus far. Some dispersal events, e.g., from East Asia to Southeast Asia in clade A and from East Asia to South Asia in clade B, are coincident with two major flyways for Anatidae in Asia [65], indicating the role of waterfowl in hydrilla distribution.

### Implications for the invasion of hydrilla

Introducing the natural enemies of weeds into their native range is an effective way to control invasive weeds. Thus, it is important to pinpoint the likely origin of invasive weeds. Two biotypes of hydrilla (dioecious and monoecious) have been recognised in the United States [32]. These biotypes are thought to have been separately introduced [35,66]. The dioecious plants were reported to have been introduced from Sri Lanka to Florida [67], and the South Asian geographic origin of the US dioecious hydrilla was confirmed in genetic studies [40,41,43]. The occurrence of the common haplotype B1/H1 in China (Figs. 1a and 2) indicates that the southern part of East Asia is also a possible original site for the US dioecious hydrilla. The monoecious plants found in the US were possibly introduced from Korea based on genetic similarity [41,43]. The occurrence of the common haplotype B4/H5 in eastern China (Figs. 1a and 2) suggests eastern China as one of the original areas for the US monoecious hydrilla. Due to the independent origin of the two biotypes of hydrilla, the search for natural enemies of hydrilla needs to be conducted in each original range, especially in the common area of China.

The northernmost monoecious hydrilla population occurs in the Lucerne/Pipe Lakes complex in Washington, at 47.37° north latitude [66]. The northernmost dioecious hydrilla population was found in Idaho but lacks detailed location information; it is most likely located at approximately 42° north latitude [40]. The two biotypes both belong to clade B in the phylogenetic tree (Fig. 2). In their native range, the hydrilla populations belonging to clade C occur farther north than the populations belonging to clade B (Fig. 1a). The northernmost population we collected occurs at 49.12° north latitude, a latitude similar to that of the US-Canada border. Based on their latitudinal distribution, hydrilla plants from clade C could easily occur in the border area between the US and Canada. Thus, importing hydrilla from the northern part of East Asia and Europe should be forbidden to avoid a new invasion of hydrilla into North America.

## Conclusions

Our study reveals that China is most likely the centre of genetic diversity in *Hydrilla*, and our findings point to an East Asian origin of *Hydrilla*. The study provides empirical evidence, based on a phylogeographic analysis, that reveals the complex biogeographic history of diversification and colonisation in worldwide species of submerged macrophytes. Our results will be more persuasive once more extensive samples from other countries are included. Comparative studies on other submerged macrophytes that are distributed worldwide would be valuable in better understanding the diversification and colonisation of this distinct group of plants.

## Methods

### Plant materials

A total of 681 individuals of hydrilla were collected at 123 sites throughout its distribution range in China, from the northeast to the southwest (Fig. 1a, Additional file 1). Three to 12 shoots per population were randomly sampled from different individuals at intervals of at least 10 m. Young, healthy plant fragments of approximately 10 cm in length were collected and dried with silica gel for subsequent DNA extraction. Voucher specimens from each population were deposited in the herbarium of Wuhan University (WH).

### DNA extraction, amplification and sequencing

Total genomic DNA was extracted from silica-dried plant fragments using the DNA Secure Plant Kit (Tiangen Biotech, Beijing, China). Primers “c” and “f” reported by Taberlet *et al.* [68] were used to amplify and sequence the chloroplast *trn*L-F region. Polymerase chain reaction (PCR) was performed using 10–30 ng of genomic DNA, 0.1 μM each primer, 0.2 mM each dNTP, 2 mM MgCl_2_, and 0.6 U of *ExTaq* DNA polymerase (TaKaRa) in a volume of 25 μL under the following conditions: 3 min at 95 °C, followed by 35 cycles of 30 s at 95 °C, 30 s at 55 °C, and 90 s at 72 °C, and then a final 5 min extension at 72 °C. Amplifications were conducted in a Veriti 96-Well Thermal Cycler (Applied Biosystems, Foster City, USA). The PCR products were purified and sequenced in both directions by the Beijing Genomic Institute in Wuhan, China. All sequences of different haplotypes were deposited in GenBank (Accession Nos. KM982392–KM982400).

### Phylogeographic analyses

Sequences were aligned using the program Mafft 6.7 [69], and manual adjustment was performed in Se-Al 2.0 [70]. The number of haplotypes (H) and polymorphic sites (S), haplotype diversity (Hd), and nucleotide diversity (Pi) were calculated using DNASP 5.10 [71]. To interpret the genealogical relationships among sequences, a median-joining network [72] based on haplotypes was generated from the cpDNA sequence data using NETWORK 4.5.1.6 (http://www.fluxus-engineering.com). In the network analysis, gaps with two or more base pairs were coded as single mutation events. When overlapping indels occurred, the overlap portion was considered a single event [73].

We defined groups of 123 populations of hydrilla based on basin boundaries. Nine groups were defined from the northeast to the southwest, corresponding to nine main basins: the Amur-Heilong River Basin (RB1), the Liao River and Hai River Basin (RB2), the Yellow River Basin (RB3), the Huai River Basin (RB4), the Yangtze River Basin (RB5), river basins in Southeast China (RB6), the Pearl River Basin (RB7), river basins in South China (RB8), and river basins in Southwest China (RB9) (Fig. 1a). An analysis of molecular variance (AMOVA) was used to partition genetic variation among and within groups, as implemented in ARLEQUIN 3.1 [74]. The occurrence of significant phylogeographic structure was tested by comparing two measures of population differentiation, G_ST_ and N_ST_, based on 1,000 permutations in PERMUT.

We examined pairwise mismatch distributions to detect historical demographic expansions using DNASP. Populations at demographic equilibrium should present a multimodal or random and rough distribution of pairwise differences, whereas populations experiencing a sudden demographic expansion are expected to display a unimodal and smooth distribution [75,76].

### Biogeographic analyses

We combined the sequences of 30 samples collected worldwide [43] into our dataset for phylogenetic analyses. Identical sequences were collapsed into a single haplotype. Based on phylogenetic studies of Hydrocharitaceae [9,77,78], three closely related species, *Vallisneria natans*, *V. spinulosa* and *Najas marina*, were included as outgroups. We conducted maximum likelihood (ML) analysis in the program GARLI [79], beginning with random trees and using 10,000,000 generations per search. Bootstrap support was estimated from 1,000 bootstrap replicates in GARLI. Bayesian inference was implemented in MrBayes 3.1.2 [80]. Two independent Markov Chain Monte Carlo (MCMC) analysis runs were conducted simultaneously, beginning with a random tree, with each run including four chains (one cold and three hot). Two million generations were run, with sampling at every 1,000 generations. Tracer 1.4 [81] was employed to check whether the chains converged, and the first 25 % of samples were discarded as burn-in. The best-fit model of nucleotide substitution for the ML and Bayesian analyses was identified under the Akaike information criterion (AIC) implemented in Modeltest 3.7 [82].

The divergence time between clades in hydrilla was estimated in two steps. First, we estimated the age of the stem and crown nodes of hydrilla based on combined 18S + *rbc*L + *mat*K + *trn*K 5’ intron + *rpo*B + *rpo*C1 + *cob* + *atp*1 sequence data from 12 genera within Hydrocharitaceae and three outgroups from Chen *et al*. [9]. We amplified and sequenced these eight fragments in four hydrilla individuals with haplotypes A2, B1, C1, and D1, representing four distinct lineages in the phylogenetic analysis (see results). All of the obtained sequences were deposited in GenBank (Accession Nos. KM982360–KM982391) and combined into the dataset. Datasets for cpDNA and 18S sequences were found to be combinable according to the incongruence length difference test [83] (*p* > 0.05). The divergence time estimate was conducted in BEAST 1.7.4 [84] using the dataset including 16 taxa. The parameter set and calibration points were the same as those used by Chen *et al*. [9]. Due to the combined dataset did not provide a topology that had nodal support within hydrilla (see Fig. 3), which was the same as any of these fragments (results not shown), we used the *trn*L-F sequences to infer the divergence time of internal nodes in hydrilla. Second, we employed the age of the stem and crown nodes of hydrilla to estimate interior divergence times in hydrilla based on the *trn*L-F sequences. We applied the GTR model of nucleotide substitution with Gamma Categories set to six under an uncorrelated lognormal relaxed clock model [85]. MCMC analyses of 300,000,000 generations were implemented, in which every 1,000 generations were sampled. The first 10 % of generations were discarded as burn-in, and the parameters were checked using the program Tracer.

Based on the Bayesian framework, three analyses were used to reconstruct the possible ancestral ranges of hydrilla. A statistical dispersal-vicariance analysis (S-DIVA) and a Bayesian binary MCMC (BBM) were implemented in the program RASP (Reconstruct Ancestral State in Phylogenies, [86]). Another event-based method dispersal-extinction cladogenesis (DEC) was implemented in the program LAGRANGE 2.0.1 [87,88]. Six areas were defined according to the distribution range of hydrilla: a) Europe (Poland); b) East Asia (China/Korea/Japan); c) Africa (Burundi); d) South Asia (India/Nepal /Pakistan); e) Southeast Asia (Vietnam/Thailand/Malaysia/Indonesia); and f) Oceania (Australia/New Zealand). Only the most closely related genus *Vallisneria* was chosen as outgroup, and its ancestral area was restricted to East Asia and Southeast Asia according to the results of Chen *et al*.[9].The invaded region of North America was excluded from these analyses due to the human introduction of hydrilla to the continent. The number of maximum areas was set to range from 2–6. In the BBM analysis, a fixed JC + G (Jukes-Cantor + Gamma) model was chosen with a null root distribution. The MCMC chains were run for 5,000,000 generations, and every 100 generations were sampled. In the DEC analysis, the dispersal probability between areas was set to the same value.

### Availability of supporting data

The data set supporting the results of this article is included within the article and its additional files. The 41 cpDNA sequences supporting the results of this article are available in the National Center for Biotechnology Information (GenBank) under accession numbers KM982360–KM982400, http://www.ncbi.nlm.nih.gov/Genbank/.

## Additional files

Additional file 1:
**Collection sites and haplotype distribution of** ***Hydrilla*** **populations in China.**
Additional file 2:
**Information of variation in the combined 18S +** ***rbc*** **L +** ***mat*** **K +** ***trn*** **K 5’ intron +** ***rpo*** **B +** ***rpo*** **C1 +** ***cob*** **+** ***atp*** **1** **sequence data and** ***trn*** **L-F sequence data for** ***Hydrilla.***
